# Diatomite-Templated Fabrication of Porous N, S-Co-Doped Carbon as High-Performance Catalyst for Oxygen Reduction

**DOI:** 10.3390/molecules31193526

**Published:** 2026-10-03

**Authors:** Qingqing Wang, Jiayi Su, Haoxiong Nan, Chenghang You

**Affiliations:** 1 School of Clinical Medicine, Hainan Vocational University of Science and Technology, Haikou 571126, China; 2 School of Chemistry and Chemical Engineering, Hainan University, Haikou 570228, China; 3 Key Laboratory of Water Pollution Treatment and Resource Reuse of Hainan Province, School of Chemistry and Chemical Engineering, Hainan Normal University, Haikou 571158, China

**Keywords:** N, S dual-doped, non-metal-based catalyst, oxygen reduction, polyacrylonitrile, diatomite

## Abstract

Oxygen reduction reaction (ORR) catalysts free of noble metals serve an essential function in boosting the practical deployment of fuel cells and metal–air batteries. Herein, an N, S-co-doped porous carbon electrocatalyst was fabricated using polyacrylonitrile (PAN), thiourea and diatomite as the precursors, sulfur source and hard template, respectively. The optimal catalyst (S-PAC-D) exhibits a high ORR performance, with a half-wave potential of 0.84 V (vs. RHE), as well as a predominant four-electron pathway, short-term stability and methanol resistance. After being constructed as a zinc–air battery (ZAB), it can deliver a peak power density of 126.6 mW·cm^−2^ and a capacity of 804.1 mAh g^−1^_Zn_. This work offers a scalable, low-cost versatile methodology to fabricate high-performance non-noble metal ORR catalysts, which holds great promise for clean energy conversion devices.

## 1. Introduction

Growing global energy scarcity and worsening ecological degradation severely hinder the sustainable progression of socioeconomic systems. Exploring high-efficiency, low-carbon, eco-friendly and low-cost energy conversion technologies has emerged as a core strategy to resolve the intertwined energy and environmental crises [1,2,3,4,5,6,7,8]. As a core electrochemical reaction for advanced energy equipment, such as proton exchange membrane fuel cells, direct methanol fuel cells, diverse metal–air batteries (e.g., lithium–air batteries, zinc–air batteries), the oxygen reduction reaction (ORR) dominates the overall energy conversion efficiency of these devices, which is highly dependent on its inherent catalytic properties and reaction kinetic characteristics [9,10,11,12,13,14,15].

Carbon materials modified with transition metal dopants are widely studied in the field of ORR catalysis due to their tunable fabrication cost and high catalytic activity. Nevertheless, these catalyst systems usually suffer from the leakage of free transition metal ions during practical service. The leaked metal ions can initiate Fenton-like chain reactions and produce strongly oxidative hydroxyl radicals [16,17]. These reactive oxidative species not only erode the internal structures of energy devices but also induce the deterioration of carbon substrates. Such undesirable issues lead to rapid attenuation of catalytic activity, making them incapable of satisfying the rigorous stability standards for long-duration practical applications.

Different from transition metal-based catalysts, metal-free carbons avoid transition metal components, thus suppressing material degradation originating from Fenton reactions [18,19,20,21]. Among single-doped carbons, nitrogen-doped carbons exhibit the highest ORR performance, although they are still undesired. To optimize ORR kinetics and practical catalytic performance before such materials can be practically implemented, more effective strategies are still required. Polyacrylonitrile (PAN) is a typical high-nitrogen polymeric precursor. Under high-temperature heat treatment, the cyano groups along the polymer chains undergo extensive cyclization and aromatization, forming a carbon framework enriched with pyridinic nitrogen, which help to retain N content and can be transformed into other ORR-active N species during the pyrolysis [22]. Therefore, PAN can be a suitable precursor for constructing high-performance metal-free carbon catalysts. Despite this, direct carbonization of pure PAN often leads to severe agglomeration, resulting in dense carbon monoliths with low porosity. Such a compact microstructure buries numerous intrinsic active sites, prevents their efficient contact with reactants, and consequently deteriorates the overall ORR catalytic performance. Therefore, pore-structure engineering can be a very robust approach to address the inherent porosity drawbacks of PAN-derived carbons, which optimizes their microstructures and mass-transport pathways.

Diatomite is a natural silica-based porous mineral, featuring rich reserves, low-cost production, environmental compatibility, and well-defined interconnected pore structures. When diatomite serves as a hard template for PAN pyrolysis, it is expected to suppress the fusion and agglomeration of polymer chains, yielding carbon materials with large specific surface areas and interlinked porous networks. These structural merits will maximize active-site exposure, optimize ion–gas transport paths, reduce interfacial mass-transfer resistance, and eventually boost catalytic performance. Additionally, existing reports have demonstrated that N, S dual-doping can deliver further ORR improvement via electronic modulation and geometric synergistic effects within carbon frameworks [23,24,25,26].

Inspired by the above considerations, we report herein a simple and scalable route for synthesizing N, S dual-doped porous carbon catalysts via a template-assisted pyrolysis method, using PAN, thiourea and diatomite as the precursors and template, respectively. The optimal catalyst (S-PAC-D) demonstrates a performance that is competitive with the commercial Pt/C catalyst. Also, the catalyst exhibits high four-electron pathway selectivity, good short-term stability and satisfactory methanol resistance. After being assembled in a zinc–air battery (ZAB), it can deliver a peak power density and a specific capacity of 126.6 mW·cm^−2^ and 804.1 mAh g^−1^_Zn_, respectively.

## 2. Results

**Figure 1:**
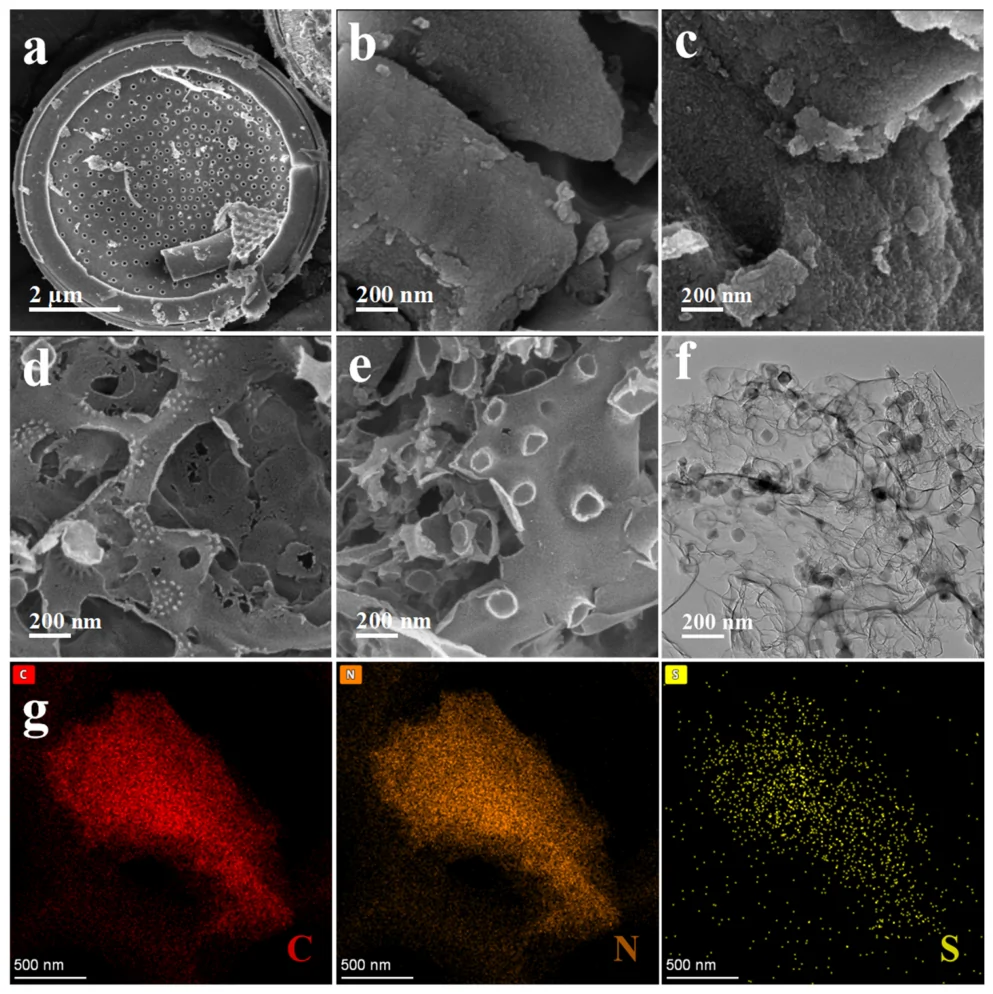
SEM images: (**a**) Diatomite; (**b**) PAC; (**c**) S-PAC; (**d**) PAC-D; and (**e**) S-PAC-D. (**f**) TEM image of S-PAC-D; (**g**) EDX mapping images for S-PAC-D.

Morphological architectures for the obtained catalytic samples were investigated via SEM and TEM techniques. As displayed in Figure 1a, raw diatomite exhibits a typical discoid morphology with abundant nanopore structures. Figure 1b shows the SEM image of the catalyst obtained by direct pyrolysis of PAN. The material displays a bulk aggregated morphology without obvious porous structure (Figure 1b), indicating that it is difficult to construct a well-developed porous structure by solely pyrolyzing PAN itself. Figure 1c illustrates the image of S-PAC, derived from the precursor containing PAN and thiourea. The morphology of S-PAC is similar to that of PAC. With diatomite incorporated as the template, the as-prepared PAC-D catalyst displays obvious sheet-like morphology, accompanied by plenty of interpenetrating channels and pores (Figure 1d). This observation proves that diatomite functions as a hard template in the pyrolysis procedure, which facilitates the generation of sheet-like carbon geometry and builds up pore frameworks. As for S-PAC-D, it displays a morphology similar to that of PAC-D (Figure 1e). The TEM image of S-PAC-D (Figure 1f) further confirms the ultrathin, wrinkled, porous carbon nanosheet structure of the material. The EDX elemental mapping results (Figure 1g) exhibit obvious C, N, and S signals over the catalyst, suggesting the homogeneous incorporation of N and S into the carbon matrix.

**Figure 2:**
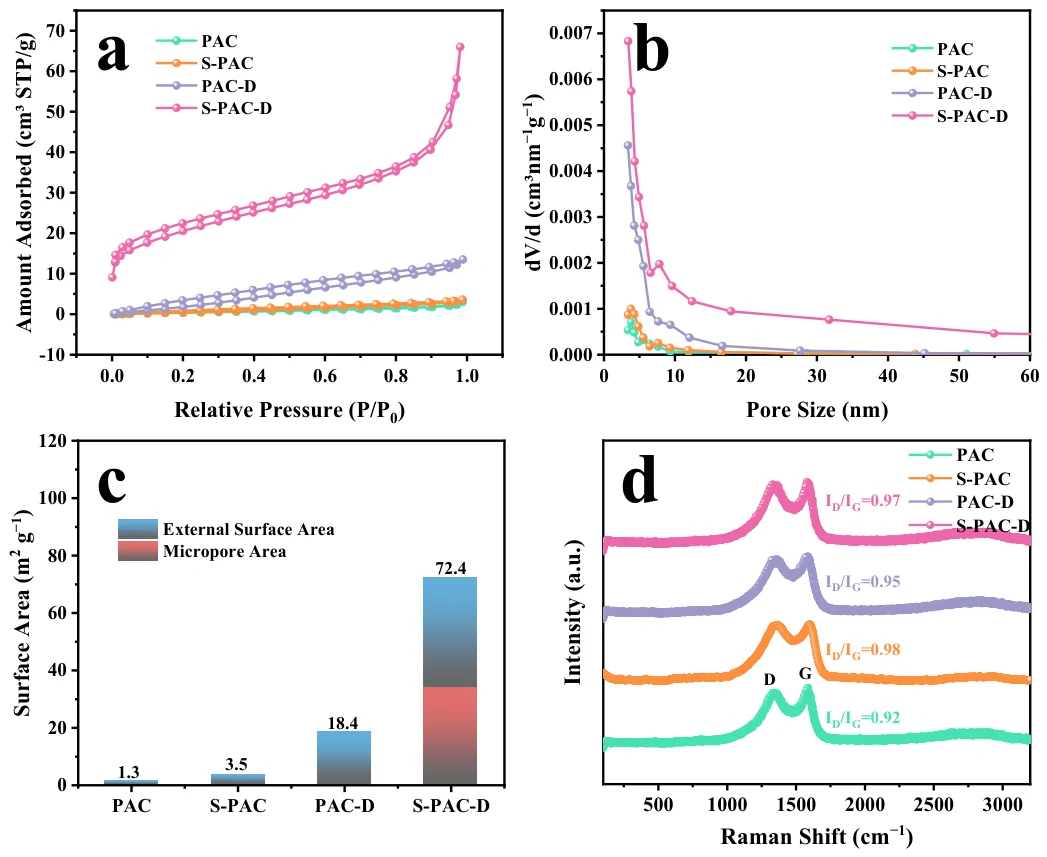
(**a**) N_2_ sorption isotherms for PAC, S-PAC, PAC-D and S-PAC-D; (**b**) pore size distribution derived from the desorption branch of N_2_ adsorption–desorption isotherms via the BJH method; (**c**) specific surface areas for obtained catalysts (t-plot results); and (**d**) Raman spectra of PAC, S-PAC, PAC-D, and S-PAC-D, the “D” and “G” represent the D-band and G-band, respectively.

To investigate the porous structure of the catalysts, we recorded their nitrogen adsorption–desorption isotherms. As can be observed in Figure 2a, the adsorption capacities of PAC and S-PAC are low in the whole-pressure regions, suggesting that PAC and S-PAC lack abundant porous structures. The nitrogen adsorption–desorption isotherms of both PAC-D and S-PAC-D exhibit typical type IV isotherm behavior, with prominent hysteresis loops, indicating that there are abundant mesoporous structures in both catalysts. In the high-pressure region, S-PAC-D shows a higher nitrogen adsorption uptake than PAC-D, indicating that S-PAC-D possesses a larger total pore volume. The pore size distribution results in Figure 2b further confirm that both materials are dominated by mesopores, and the pore volume and pore size distribution intensity of S-PAC-D are much higher than those of PAC-D. The Brunauer–Emmett–Teller (BET) surface area data in Figure 2c shows that PAC-D has a specific surface area of 18.4 m^2^g^−1^, whereas S-PAC-D reaches 72.4 m^2^g^−1^, the highest among all the catalysts. A comparison of the t-plot results for PAC-D and S-PAC-D (Figure 2c) reveals that the specific surface area of PAC-D originates almost entirely from the external surface, with micropores contributing negligibly. In contrast, the micropore structure in S-PAC-D contributes 34.0 m^2^g^−1^ to the specific surface area, accounting for nearly half of the total specific surface area, which demonstrates abundant micropores in S-PAC-D. Evidently, these micropores in S-PAC-D arise from the introduction of thiourea. In addition to micropore area, the external surface area of S-PAC-D also reaches 38.4 m^2^g^−1^, more than twice that of PAC-D, indicating that thiourea incorporation not only constructs microporous structures within the catalyst but also effectively increases the proportion of mesopores. These optimized porous structures are expected to smooth the mass transfer and improve the accessibility of the inner active sites, and eventually enhance the ORR performance.

To explore the graphitic degree and defect density of the resultant catalysts, Raman spectroscopy was used. As summarized in Figure 2d, the I_D_/I_G_ value of PAC is 0.92, indicating a relatively low defect density due to the dense, bulk carbon structure formed by direct pyrolysis of solely PAN. After introducing the diatomite template, the I_D_/I_G_ value of PAC-D increases to 0.95, which can be attributed to the structural distortion and edge defects generated during the formation of the sheet-like porous architecture. In contrast, sulfur incorporation plays a more prominent role in defect generation: the I_D_/I_G_ value of S-PAC reaches 0.98, the highest among all the catalysts, demonstrating that thiourea-mediated sulfur doping can introduce abundant structural defects into the carbon matrix. Notably, the I_D_/I_G_ ratio of S-PAC-D (0.97) is slightly lower than that of S-PAC but remains markedly higher than those of PAC and PAC-D, suggesting that the combination of sulfur doping and diatomite templating preserves the high defect density while simultaneously constructing a porous architecture.

**Figure 3:**
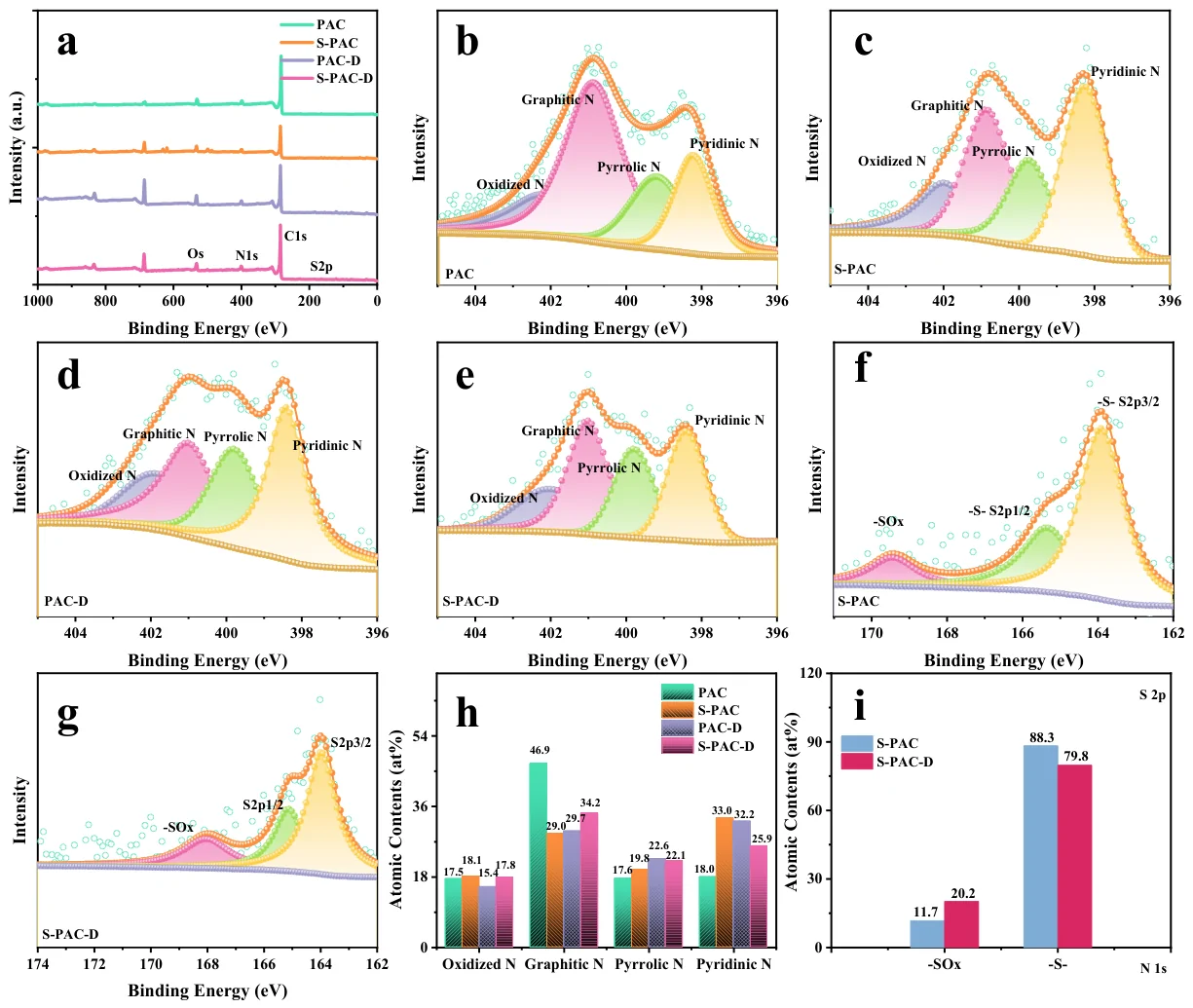
(**a**) XPS spectra of obtained catalysts; high-resolution N 1s spectra: (**b**) PAC; (**c**) S-PAC; (**d**) PAC-D; (**e**) S-PAC-D; high-resolution S 2p spectra: (**f**) S-PAC; (**g**) S-PAC-D; (**h**) atomic composition for different N species in obtained catalysts; and (**i**) atomic composition for S species in S-PAC-D. Peaks with different colors represent the fitted sub-peaks of N and S species.

**Figure 4:**
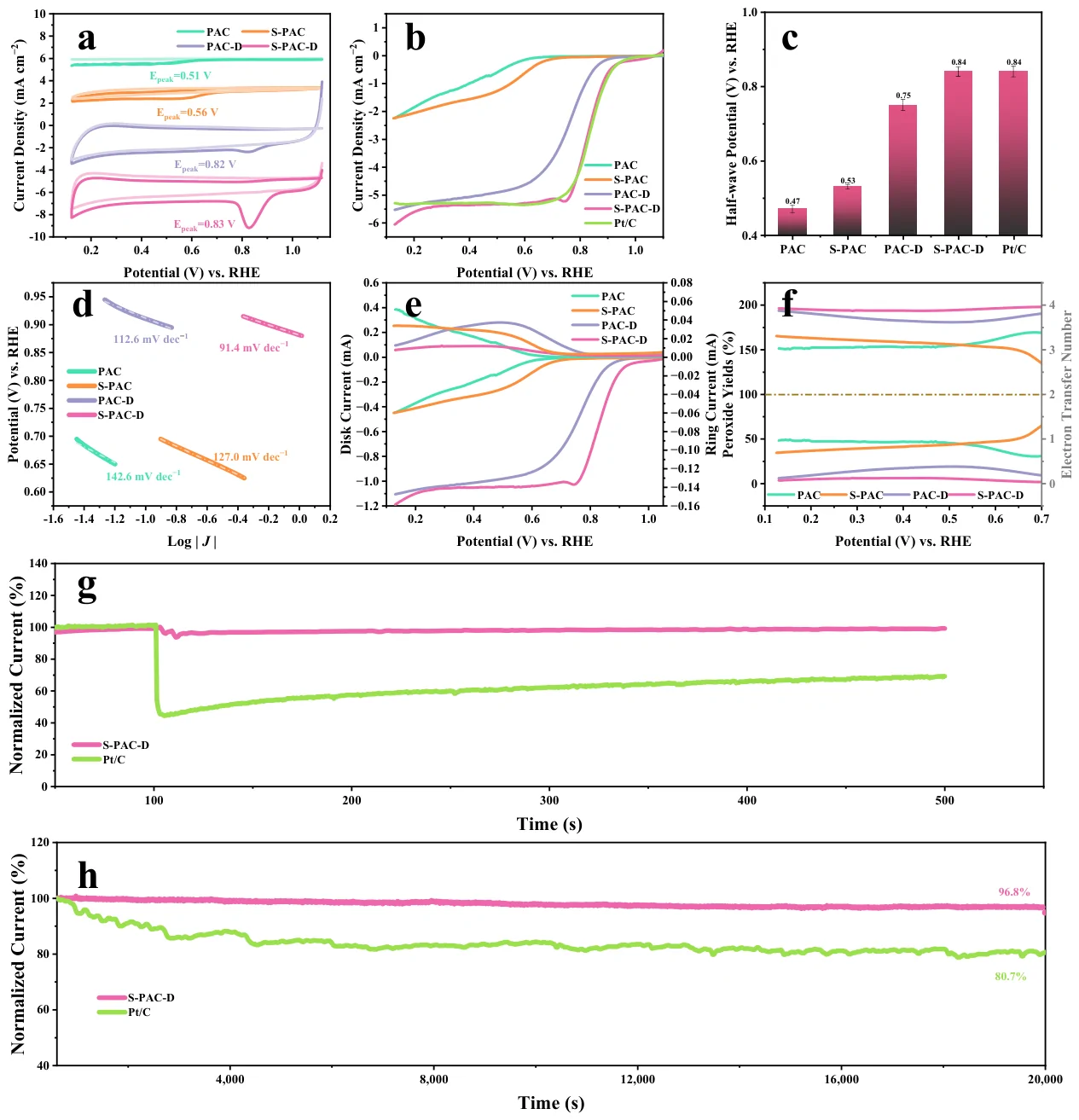
(**a**) CV curves; (**b**) ORR LSV polarization curves for obtained catalysts; (**c**) half-wave potentials (E_1/2_); (**d**) Tafel analysis; (**e**) RRDE test results; (**f**) peroxide yield and electron-transfer number for different catalysts; (**g**) methanol tolerance measurement results; and (**h**) durability test results.

Figure 3a–e present the high-resolution X-ray photoelectron spectroscopy (XPS) characterization results of different samples. The quantitative analysis of nitrogen species (Figure 3f) reveals that PAC is dominated by graphitic-N (46.9 at.%), while the relative contents of pyridinic-N and pyrrolic-N are only 18.0 and 17.6 at.%, respectively. After introducing the diatomite template, the graphitic-N content of PAC-D decreases markedly to 29.7 at.%, whereas the relative contents of pyridinic-N and pyrrolic-N increase to 32.2 and 22.6 at.%, respectively. A very similar trend is also observed for S-PAC upon the addition of thiourea alone: its graphitic-N content drops from 46.9 at.% of PAC to 29.0 at.%, accompanied by a substantial rise in pyridinic-N from 18.0 to 33.0 at.% and an increase in pyrrolic-N from 17.6 to 19.8 at.%. These results indicate that both the diatomite template and sulfur incorporation can effectively convert graphitic-N into pyridinic-N and pyrrolic-N. The oxidized-N content stays almost unchanged among all the catalysts (15.4–18.1 at.%), suggesting that the template and thiourea mainly regulate the distribution of active nitrogen species rather than the oxidized fraction. Since pyridinic-, pyrrolic- and graphitic-N are all recognized as high-activity sites for ORR, S-PAC-D is expected to be more ORR-active than the others, which will be discussed in the next section (Figure 4).

The high-resolution S 2p spectra (Figure 3e) confirm that sulfur was successfully doped into the carbon skeleton in the form of covalent bonds in both S-PAC and S-PAC-D. The quantitative results of sulfur species in Figure 3f show that 88.3 at.% of the sulfur in S-PAC is -S-, with only 11.7 at.% being oxidized sulfur. For S-PAC-D, 79.8 at.% of sulfur remains in the form of thiophene sulfur, while the oxidized sulfur fraction rises slightly to 20.2 at.%. Nevertheless, the predominant thiophene sulfur in both sulfur-doped catalysts indicates that most of the sulfur survived pyrolysis in a highly active form, which has been reported to influence the electrocatalytic performance of carbon catalysts [23,24,25,26].

### Electrochemical Characterization

Figure 4 displays the electrocatalytic ORR performance of the resultant carbon-based catalysts. As shown in the cyclic voltammetry (CV) curves (Figure 4a), all catalysts exhibit distinct cathodic peaks in O_2_-saturated electrolyte, whereas no corresponding peak is observed in the N_2_-saturated control curves, confirming that the cathodic response originates from ORR. The ORR peak potentials follow the order of PAC (0.51 V) < S-PAC (0.56 V) < PAC-D (0.82 V) < S-PAC-D (0.83 V). The linear sweep voltammetry (LSV) polarization curves (Figure 4b,c) further show that the half-wave potential of S-PAC-D reaches 0.84 V, higher than those of PAC (0.47 V), S-PAC (0.53 V) and PAC-D (0.75 V) and comparable to that of commercial Pt/C (0.84 V), and which surpasses recently reported metal-free ORR catalysts (Table S2). In addition, S-PAC-D delivers the highest limiting current density among the series. These results indicate that S-PAC-D is the most ORR-active among the four catalysts.

The Tafel slope of S-PAC-D (91.6 mV dec^−1^, Figure 4d) is lower than those of PAC (142.6 mV dec^−1^), S-PAC (127.0 mV dec^−1^) and PAC-D (112.6 mV dec^−1^), indicating the most favorable ORR kinetics of S-PAC-D. The lower Tafel slopes of S-PAC and PAC-D relative to PAC suggest that both the thiourea and diatomite introductions enhance the ORR kinetics of the catalysts, which may be associated with the pore-structure regulation by thiourea and the diatomite template. The rotating ring-disk electrode (RRDE) results (Figure 4e,f) show that, over the potential range of 0.2–0.7 V, the peroxide yield of S-PAC-D remains below 5% with a corresponding electron-transfer number (*n*) of 3.90–3.95, indicating that the ORR on S-PAC-D proceeds predominantly through a four-electron transfer pathway. In contrast, the electron-transfer number of S-PAC decreases from ~3.5 to ~2.8 with increasing potential, indicating an increased contribution of the two-electron pathway in the absence of the porous template, while S-PAC-D maintains high four-electron selectivity throughout the whole potential window. In addition, the current of S-PAC-D remains essentially unchanged after methanol injection, whereas that of commercial Pt/C drops immediately (Figure 4g), indicating the better methanol tolerance of S-PAC-D. The chronoamperometric test results show that S-PAC-D retains 96.8% of its initial current after 20,000 s (~5.6 h) of continuous operation, higher than that of Pt/C (80.7%) under the same conditions (Figure 4h), demonstrating the favorable short-term stability of S-PAC-D.

**Figure 5:**
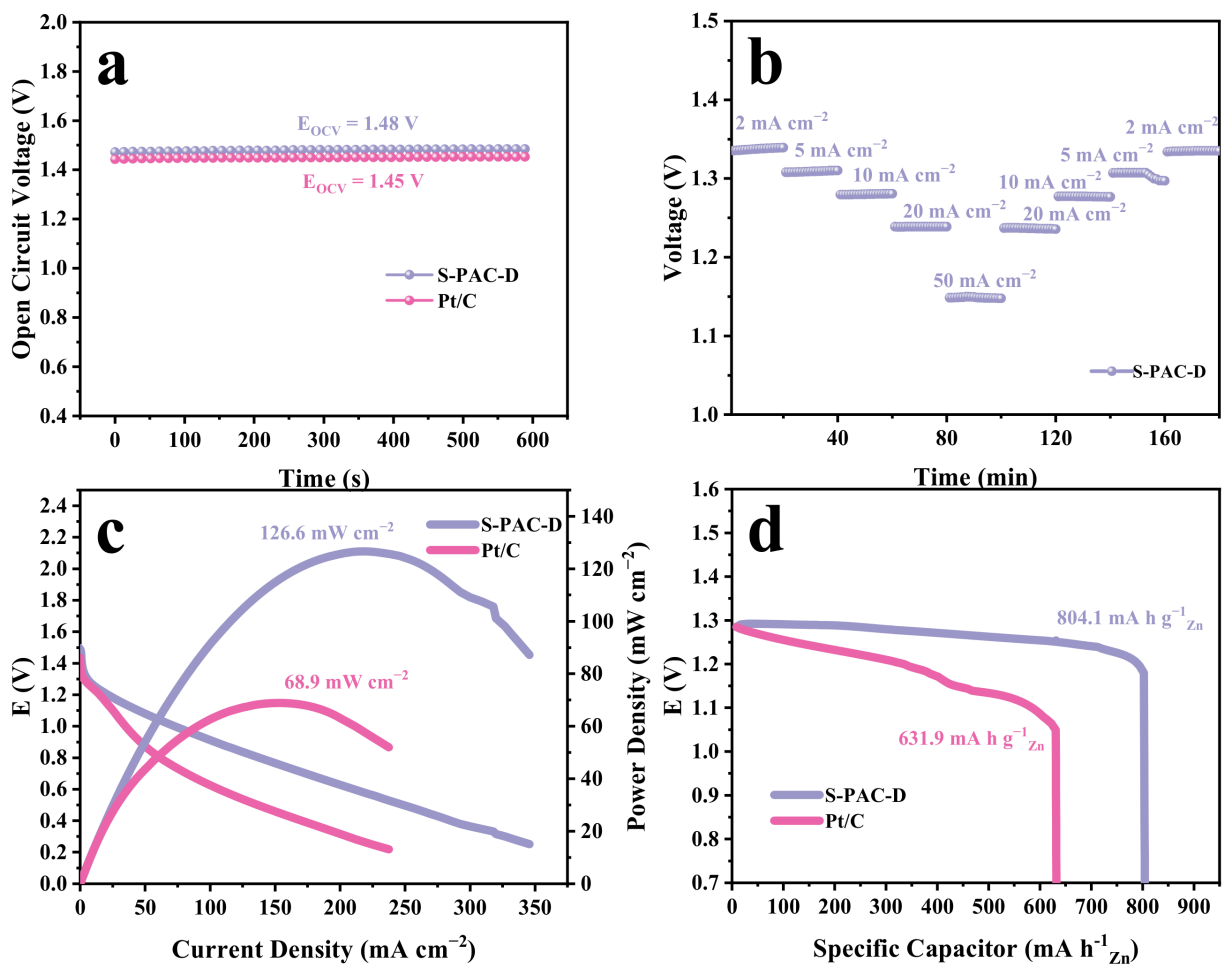
(**a**) OCV of ZABs based on S-PAC-D and Pt/C; (**b**) stepped-current galvanostatic discharge curves; (**c**) discharge and power density plots of various ZABs; and (**d**) full discharge profiles and their corresponding specific capacities.

To further assess the real-device electrocatalytic performance, we assembled the S-PAC-D catalyst into a ZAB. S-PAC-D-based ZAB shows an open-circuit voltage (OCV) value of 1.48 V (Figure 5a), surpassing 1.45 V of the battery assembled with the commercial Pt/C catalyst. Also, S-PAC-D-based ZABs exhibit stable galvanostatic discharge profiles at diverse current densities, confirming their good electrochemical reversibility (Figure 5b) and tolerance to variable operating conditions. The discharge curve results indicate that the voltage drop of the S-PAC-D-based battery during discharge is obviously smaller than that of the Pt/C-based counterpart. Also, S-PAC-D-based ZAB achieves a peak power density of 126.6 mW cm^−2^, surpassing the 68.9 mW cm^−2^ obtained for the Pt/C-based ZAB (Figure 5c). Upon complete discharge, the capacity of the S-PAC-D-based battery reaches 804.1 mAh g^−1^_Zn_, markedly exceeding the Pt/C counterpart (631.9 mAh g^−1^_Zn_), demonstrating that S-PAC-D catalyst delivers better practical discharge performance compared with commercial Pt/C (Figure 5d).

## 3. Materials and Methods

### 3.1. Materials

DMF, thiourea, PAN, diatomite and potassium hydroxide (KOH) were acquired from Macklin Co., Ltd. (Shanghai, China). Gas diffusion layer (GDL, Sigracet-28BC, Shanghai, China) was supplied by SGL Carbon SE (Wiesbaden, Germany). Carbon powder (Ketjenblack EC-300J, ~800 m^2^ g^−1^) was purchased from Lion Specialty Chemicals Co., Ltd. (Tokyo, Japan). All raw reagents were of analytical purity grade and used directly without further purification or pretreatment. Data fitting and graph plotting were performed using XPS Peak 4.1 and OriginPro 2024.

### 3.2. Catalyst Fabrication

**Figure 6:**
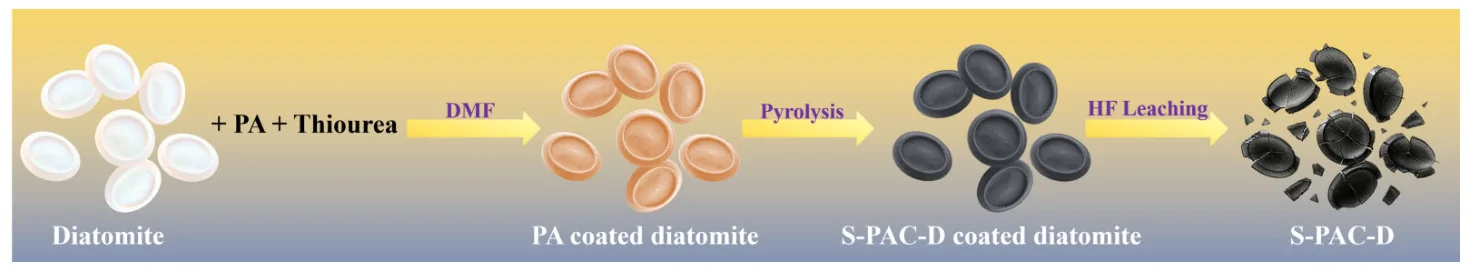
Schematic representation of the catalyst fabrication procedure.

In total, 0.25 g polyacrylonitrile (PAN) was first mixed with 1.0 g thiourea in 100 mL of N,N-dimethylformamide (DMF). The mixture was continuously stirred until both PAN and thiourea were dissolved (Figure 6). Subsequently, 1.0 g of diatomite was added, and the suspension was kept at 120 °C for 20 min under stirring. After that, the obtained mixture was heated at 130 °C to remove DMF, and the powdery solid at the bottom was collected. The powdery solid was heated in a nitrogen atmosphere and pyrolyzed at 900 °C for 1 h. After that, the obtained product was first leached in hydrofluoric acid. The solid obtained after centrifugation underwent washing using water, followed by vacuum-drying to afford the catalyst S-PAC-D, where “S” indicates that the catalyst contains sulfur, while “D” means that diatomite was used in the preparation process of the catalyst. For comparison, a sample without diatomite and thiourea (PAC), a sample using only PAN and diatomite (PAC-D), and a sample using only PAN and thiourea were prepared through similar processes.

### 3.3. Working Electrode Fabrication

A 5 mm diameter glassy carbon electrode (GCE) was employed as the working electrode support. Prior to each test, the GCE was ultrasonically rinsed with ethanol, carefully polished with 50 nm α-Al_2_O_3_ slurry on microfiber cloth, and thoroughly rinsed with deionized water to obtain a clean and smooth electrode surface. The catalyst dispersion was formulated via dispersing 5.0 mg of catalyst powder into 1 mL of 0.25 wt% Nafion-ethanol solution, accompanied by 30 min sonication treatment. The as-obtained catalyst dispersion (20 μL) was uniformly drop-cast on the pretreated GCE and dehydrated under infrared irradiation (catalyst loading: 0.5 mg cm^−2^). To evaluate the ORR catalytic activity comparatively, a commercial 20 wt% Pt/C benchmark catalyst purchased from Johnson Matthey Corp. (Royston, UK) was utilized as the reference material, where the Pt loading was calibrated to 0.1 mg cm^−2^.

### 3.4. Air Electrode Fabrication

The catalyst ink for the air electrode for ZAB was the same as the one used for the working electrode preparation. 100 μL of the prepared slurry was uniformly painted onto a gas diffusion layer (GDL, SIGRACET), prior to drying via infrared light exposure (catalyst loading 0.5 mg cm^−2^).

### 3.5. Structural Characterizations

The morphological characteristics and nanostructural information of the as-fabricated catalyst samples were systematically characterized via SEM and TEM techniques. Field-emission SEM measurements (acceleration voltage: 5 kV) were carried out on a JSM-7100F instrument (JEOL, Akishima, Japan). TEM micrographs (working voltage: 200 kV) were collected by a JEM-2100 electron microscope (JEOL, Akishima, Japan). Surface element distribution and chemical valence states for the obtained catalyst materials and precursors were analyzed via XPS measurements on an ESCALAB 250 spectrometer (Thermo-VG Scientific, Waltham, MA, USA) with monochromatic Al Kα irradiation. Nitrogen adsorption–desorption tests performed at 77 K on the Tristar II 3020 (Micromeritics, Norcross, GA, USA) were applied to analyze the specific surface area and pore-structure features. A LabRAM HR Evolution spectrometer (HORIBA, Palaiseau, France) was utilized to record Raman spectra (532 nm).

### 3.6. Electrochemical Analyses

The electrocatalytic ORR behaviors of these samples were measured with a typical three-electrode test system. The Hg/HgO (1 M KOH) electrode from Gaoss Union (Tianjin, China) was chosen as the reference, and a graphite rod served for the counter electrode. Prior to testing, 0.1 M KOH aqueous solution was bubbled with high-purity O_2_ (99.999%) for no less than 30 min to establish an oxygen-saturated testing environment. All electrochemical tests were implemented on a CHI760D analyzer (CHI, Suzhou, China) combined with an RRDE assembly (Gamry, Warminster, PA, USA).

All original potentials were calibrated to the reversible hydrogen electrode (RHE) scale by means of the Nernst equation:

E_(RHE)_ = E_(Hg/HgO)_ + 0.0592 × pH + 0.098

All acquired current densities were normalized against the geometric area of the glassy carbon electrode (0.1964 cm^2^).

RRDE tests were implemented in 0.1 M KOH solution (1600 rpm). The Pt ring was fixed at 1.42 V (vs. RHE). The hydrogen peroxide yield (*η*) and average electron transfer number (*n*) during ORR were calculated via the following formulas:

*η* = 200*I_r_* (N*I_d_* + *Ir*)^−1^

n = 4*I_d_* (*I_d_* + *I_r_*N^−1^)^−1^

where *I_r_* stands for the ring-electrode current, *I_d_* denotes the disk-electrode current, and N is defined as the collection efficiency calibrated to 0.36 using the K_3_Fe(CN)_6_ redox couple.

Chronoamperometry was further conducted at 0.72 V (vs. RHE) to evaluate the catalyst’s methanol-resistance and electrochemical durability.

### 3.7. Zinc–Air Battery Measurements

Battery performance tests were conducted using a home-made ZAB device. A mixed solution (6 M KOH + 0.2 M zinc acetate) was applied as the electrolyte, with a zinc plate functioning as the anode electrode. Galvanostatic cyclic tests were implemented on a CT3004A analyzer (LANHE, Wuhan, China).

## 4. Conclusions

In summary, a simple and effective synthetic route is herein reported to fabricate N, S-dual-doped porous carbon electrocatalysts, using polyacrylonitrile (PAN), thiourea and diatomite as the precursors, sulfur sources and hard template, respectively. The diatomite template effectively inhibits PAN agglomeration during carbonization, constructing a porous structure that enhances active-site exposure and mass transport. The optimal catalyst (S-PAC-D) achieves performance competitive against commercial Pt/C benchmarks, along with a near-ideal four-electron ORR pathway, short-term stability and satisfactory methanol resistance. After being constructed as a ZAB, a peak power density reaching 126.6 mW·cm^−2^ and a specific capacity of 804.1 mAh g^−1^_Zn_ are obtained. This work offers a scalable, low-cost versatile methodology to fabricate high-performance non-noble metal ORR catalysts.

## Data Availability

The original contributions presented in this study are included in the article/Supplementary Materials. Further inquiries can be directed to the corresponding authors.

## Supplementary Materials

The following supporting information can be downloaded at: https://www.mdpi.com/article/10.3390/molecules31193526/s1, Figure S1. Si contents in S-PAC-D before and after HF leaching. Table S1. t-plots results based on the N_2_ adsorption-desorption isotherms. Table S2. comparison of S-PAC-D with other metal-free ORR catalysts recently reported. Refs. [27,28,29,30,31,32,33,34,35] are cited in Supplementary Materials.
