# Supplementary material for: Diatomite-Templated Fabrication of Porous N, S-Co-Doped Carbon as High-Performance Catalyst for Oxygen Reduction
Source: Molecules. 2026 Oct 3;31(19):3526. doi: 10.3390/molecules31193526 (PMC13667131; doi:10.3390/molecules31193526)
Supplement: Supplementary file 1 [file molecules-31-03526-s001.zip › molecules-4517040-supplementary.pdf]

Supporting Information

Diatomite-Templated Fabrication of Porous N, S-Co-Doped Carbon as High-Performance Catalyst for Oxygen Reduction

Qingqing Wang [1], Jiayi Su [1], Haoxiong Nan [2,*] and Chenghang You [3,*]

[1] School of Clinical Medicine, Hainan Vocational University of Science and Technology, Haikou 571126, China
[2] School of Chemistry and Chemical Engineering, Hainan University, Haikou 570228, China
[3] Key Laboratory of Water Pollution Treatment and Resource Reuse of Hainan Province, School of Chemistry and Chemical Engineering, Hainan Normal University, Haikou 571158, China
* Correspondence: nanhaoxiong@hainanu.edu.cn (H.N.); you.ch@hainnu.edu.cn (C.Y.)

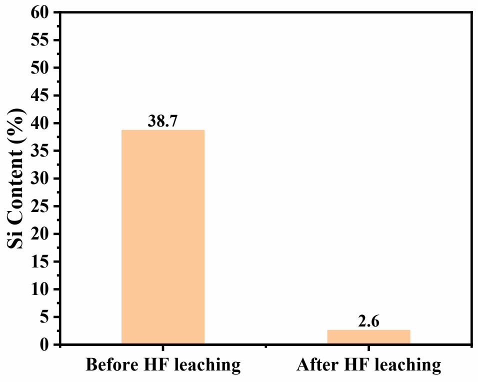

Figure S1. Si contents in S-PAC-D before and after HF leaching

Table S1. t-plots results based on the N_2 adsorption-desorption isotherms

	Micropore volume/cm^3g^{-1}	Micropore area/$m^2 g^{-1}$	External surface area/$m^2 g^{-1}$	Average pore diameter/nm	Total pore volume/cm^3g^{-1}
PAC	0	0	1.3	1.4	0.0046
S-PAC	0	0	3.5	6.3	0.0056
PAC-D	0	0	18.4	4.6	0.0210
S-PAC-D	0.0173	34.0	38.4	5.6	0.1023

Table S2. comparison of S-PAC-D with other metal-free ORR catalysts recently reported.

No.	Catalysts	$E_{1/2}$ (vs. RHE)	Electrolyte	References
1	CTS/Cl	0.82	0.1 M KOH	[1]
2	N,P-C-1000)	0.83	0.1 M KOH	[2]
3	DAF-COF	0.74	0.1 M KOH	[3]
4	NR-900	0.837	0.1 M KOH	[4]
5	SAN/PAN/CNT-1100	0.72	0.1 M KOH	[5]
6	N, F-MCFs	0.81	0.1 M KOH	[6]
7	NSGD-800	0.754	0.1 M KOH	[7]
8	PYTA-PB-C	0.78	0.1 M KOH	[8]
9	BUCT-COF	0.72	0.1 M KOH	[9]
10	JUC-616	0.78	0.1 M KOH	[10]
	**S-PAC-D**	**0.84**	**0.1 M KOH**	**Our work**

References

1. Lin, C.; Cao, X.; Wang, L.; Cai, M.; Ma, R.; Ma, J., S/Cl Co-doped biomass-derived carbon electrocatalysts: Low △E of 0.58 V for ORR/OER and application in high-performance zinc-air batteries. *Nano Energ.* **2026,** 150, 111791.
2. Ao, K.; Yue, X.; Zhang, X.; Zhao, H.; Liu, J.; Shi, J.; Daoud, W. A.; Li, H., N-P covalent bond regulation of mesoporous carbon-based catalyst for lowered oxygen reduction overpotential and enhanced zinc-air battery performance. *Journal of Colloid and Interface Science* **2024,** 672, 107-116.
3. You, Z.; Wang, B.; Zhao, Z.; Zhang, Q.; Song, W.; Zhang, C.; Long, X.; Xia, Y., Metal-Free Carbon-Based Covalent Organic Frameworks with Heteroatom-Free Units Boost Efficient Oxygen Reduction. *Adv. Mater.* **2023,** 35, (7), 2209129.
4. Srinivas, K.; Liu, D.; Ma, F.; Chen, A.; Zhang, Z.; Wu, Y.; Wu, Q.; Chen, Y., Defect-Engineered Mesoporous Undoped Carbon Nanoribbons for Benchmark Oxygen Reduction Reaction. *Small* **2023,** 19, (34), 2301589.
5. Mooste, M.; Kibena-Põldsepp, E.; Vassiljeva, V.; Merisalu, M.; Kook, M.; Treshchalov, A.; Kisand, V.; Uibu, M.; Krumme, A.; Sammelselg, V.; Tammeveski, K., Electrocatalysts for oxygen reduction reaction based on electrospun polyacrylonitrile, styrene‑acrylonitrile copolymer and carbon nanotube composite fibres. *Journal of Materials Science* **2019,** 54, (17), 11618-11634.
6. Gong, T.; Qi, R.; Liu, X.; Li, H.; Zhang, Y., N, F-Codoped Microporous Carbon Nanofibers as Efficient Metal-Free Electrocatalysts for ORR. *Nano-Micro Letters* **2019,** 11, (1), 9.
7. Huo, M.; Sun, J.; Liu, W.; Li, Q.; Chang, J.; Xing, Z., Sulfur and nitrogen dual-doped graphdiyne as a highly efficient metal-free electrocatalyst for the Zn-air battery. *Science China Materials* **2024,** 67, (12), 4005-4012.
8. Yang, X.; Fu, Y.; Liu, M.; Zheng, S.; Li, X.; Xu, Q.; Zeng, G., Solvent Effects on Metal-free Covalent Organic Frameworks in Oxygen Reduction Reaction. *Angew. Chem. Int. Ed. Engl.* **2024,** 63, (16).
9. Bao, R.; Xiang, Z.; Qiao, Z.; Yang, Y.; Zhang, Y.; Cao, D.; Wang, S., Designing Thiophene-Enriched Fully Conjugated 3D Covalent Organic Framework as Metal-Free Oxygen Reduction Catalyst for Hydrogen Fuel Cells. *Angew. Chem. Int. Ed. Engl.* **2023,** 62, (4).
10. Li, J.; Jia, J.; Suo, J.; Li, C.; Wang, Z.; Li, H.; Valtchev, V.; Qiu, S.; Liu, X.; Fang, Q., Metal-free

covalent organic frameworks containing precise heteroatoms for electrocatalytic oxygen reduction reaction. *Journal of Materials Chemistry A* **2023,** 11, (34), 18349-18355.
